# Biomechanical Properties of Insect Wings: The Stress Stiffening Effects on the Asymmetric Bending of the *Allomyrina dichotoma* Beetle's Hind Wing

**DOI:** 10.1371/journal.pone.0080689

**Published:** 2013-12-05

**Authors:** Ngoc San Ha, Quang Tri Truong, Nam Seo Goo, Hoon Cheol Park

**Affiliations:** Biomimetics and Intelligent Microsystem Laboratory, Department of Advanced Technology Fusion, Konkuk University, Seoul, Republic of Korea; University of Tours, France

## Abstract

Although the asymmetry in the upward and downward bending of insect wings is well known, the structural origin of this asymmetry is not yet clearly understood. Some researchers have suggested that based on experimental results, the bending asymmetry of insect wings appears to be a consequence of the camber inherent in the wings. Although an experimental approach can reveal this phenomenon, another method is required to reveal the underlying theory behind the experimental results. The finite element method (FEM) is a powerful tool for evaluating experimental measurements and is useful for studying the bending asymmetry of insect wings. Therefore, in this study, the asymmetric bending of the *Allomyrina dichotoma* beetle's hind wing was investigated through FEM analyses rather than through an experimental approach. The results demonstrated that both the stressed stiffening of the membrane and the camber of the wing affect the bending asymmetry of insect wings. In particular, the chordwise camber increased the rigidity of the wing when a load was applied to the ventral side, while the spanwise camber increased the rigidity of the wing when a load was applied to the dorsal side. These results provide an appropriate explanation of the mechanical behavior of cambered insect wings, including the bending asymmetry behavior, and suggest an appropriate approach for analyzing the structural behavior of insect wings.

## Introduction

For many years, insect flight has attracted significant attention in science and engineering since the concept of a micro aerial vehicle, inspired by insect flight, has become of interest [1]–[9]. During flight, an insect wing deforms significantly (bending and twisting) as it flaps. Insect wing deformation can vary greatly from stroke to stroke [10], inducing thrust asymmetry between half-strokes; thus, wing deformation has an important function in the enhancements of thrust and lift throughout a stroke cycle [11].

The bending asymmetry of insect wings is well known and has been reported numerous times in the literature based on experimental results. For example, Lehmann *et al.* [12] found that a blowfly's wing bent more easily when pushed on the dorsal side than on the ventral side through experiments. The dorsal-ventral bending asymmetry has also been shown in the wings of butterflies [13] and hawk moth *Manduca sexta* [14], and in the hind wings of locusts [15]. However, a comprehensive understanding of the structural origin of the bending asymmetry remains unclear. Therefore, another method is required to reveal the underlying theory behind the experimental results.

The finite element method (FEM) approach is a powerful tool for evaluating experimental measurements and it is widely used to study the static and dynamic structural behaviors of insect wings. The FEM has been used to model the wings of dragonflies [16], [17]. Herbert *et al*. [18] and Wootton *et al*. [19] combined suitable materials and suitable geometric properties in an FEM model of a locust wing in order to study the “umbrella effect”, which is a mechanism of camber generation in the hind wing fans of *orthopteroid* and *dictyopterroid* insects. Combes and Daniel [14], [20] investigated the effect of wing venation on the flexural stiffness of *Manduca sexta* wings and the influence of different spatial patterns of flexural stiffness on the bending behavior of the wings. Wootton *et al*. [19] and Jongerius *et al*. [21] performed a modal analysis on a slightly cambered, simplified model of *Manduca sexta* wings and dragonfly wings in order to determine the natural frequencies of their wings. However, the FEM has rarely been used to understand the bending asymmetry in insect wings, even though Combes and Daniel [14] mentioned and explored the effects of camber on bending asymmetry using a finite element model of a *Manduca sexta* wing. Unfortunately, they did not locate the bending asymmetry in the wing using the FEM. It seems that Combes and Daniel could not locate the bending asymmetry because their FEM model did not consider the nonlinear effects of a large deformation; in particular, the stress stiffening due to the large deflection compared to the thickness of the wing. The stress stiffening effect demonstrated that the out-of-plane stiffness of a structure could be significantly influenced by the in-plane stress in that structure [22]. This coupling between the in-plane stress and transverse stiffness is most prominent in thin, highly stressed structures such as wing membranes [16].

Recently, the *Allomyrina dichotoma* beetle, which has many interesting features for mimicking flapping-wing micro air vehicles (FW-MAVs), has been studied. The flapping frequency of the *Allomyrina dichotoma* beetle ranges from 30 to 40 Hz, which is not high compared with those of other insects. Moreover, these beetles, which are one of the largest insect species, have a relatively high load carrying capacity (capable of flying with an additional load of 20–30% body weight) [23]. Therefore, they are often selected to realize insect cyborgs [23] and FW-MAVs [24]–[27]. The aerodynamic characteristics of the beetle have been investigated using numerical simulations [28] and Particle Image Velocimetry (PIV) [29]. Through observations, it was found that the *Allomyrina dichotoma* beetle's hind wing exhibits bending asymmetry during free flight [30].

In this study, we considered the nonlinear effects of a large deformation that included stress stiffening in the FEM model in order to investigate the bending asymmetry in an insect wing. We created five different finite element models of the *Allomyrina dichotoma* beetle's hind wing in order to investigate the stressed stiffening effect of a membrane and the camber effects on the asymmetric bending of a wing structure: no-camber model, chordwise camber intact model, spanwise camber intact model, chord-spanwise camber intact model, and chord-spanwise camber cutting model.

## Materials and Methods

### Ethics statement

No specific permits were required for the described field study. The insects collected did not involve endangered or protected species.

### Morphology

**Figure 1:**
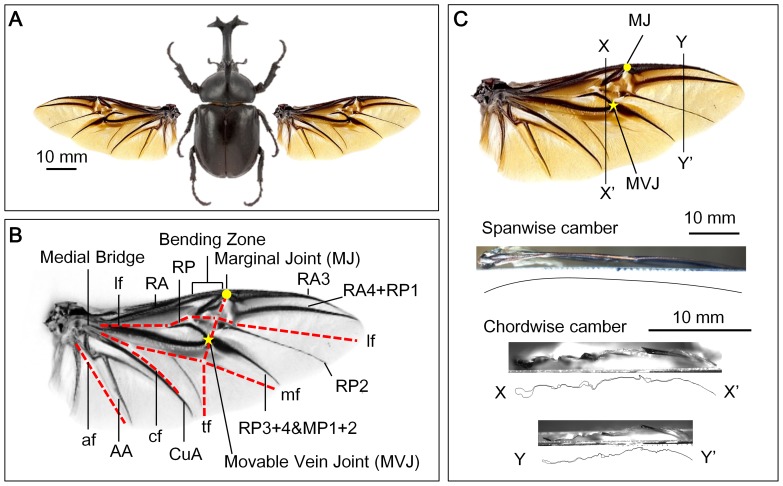
Morphology of *Allomyrina dichotoma* beetle's hind wing. (A) The *Allomyrina dichotoma* beetle and its flexible hind wing, and (B) its right hind wing showing veins. (AA, anal anterior; CuA, cubitus anterior; af, anal fold; cf, claval fold; lf, longitudinal fold; mf, median flexion line; MP1+2, media posterior; AP3+4, anal posterior; RA, radius anterior; RP, radius posterior; ScA, subcosta anterior; tf, transverse fold; the circle denotes the marginal joint (MJ) position and the star denotes the movable vein joint (MVJ) position.). (C) Spanwise and chordwise camber of the wing at the rest condition.

The *Allomyrina dichotoma* beetle was investigated in this study (see Figure 1A). This beetle was bought from a local company in South Korea. As shown in Figure 1B, the morphology of the *Allomyrina dichotoma* beetle's hind wing is similar to that of the *Pachnoda marginata* beetle [31]. The radius anterior (RA) is ﬂexible and has a bending zone and a marginal joint (MJ) at approximately half the wing length. The radius posterior (RP) 3+4 forms an articulation with the media posterior (MP) 1+2, called a movable vein joint (MVJ), where the transverse fold (tf) crosses. It is clear that the membrane, especially near the wing root and leading edge, has deep grooves between the longitudinal veins. Furthermore, the anal anterior (AA), cubitus anterior (CuA), and radius posterior (RP) veins are ventrally deflected. Through observations, when the wing is folded and shielded beneath the elytra, the hind wing lies curled on the thorax surface, which is curved. Therefore, most veins are bent at rest. Consequently, the hind wing is cambered in both chordwise and spanwise directions (see Figure 1C).

### Finite element modeling

In order to investigate how the camber and stress stiffening of the membrane affect the asymmetric bending, we used the commercial software ANSYS^®^ (v.12, ANSYS, USA) to create a simplified finite element model of the hind wing of a male beetle instead of a real model. We created five finite element models of the wing in order to investigate the effects of the camber and stress stiffening on the asymmetric bending. The five models are described as follows.

**Figure 2:**
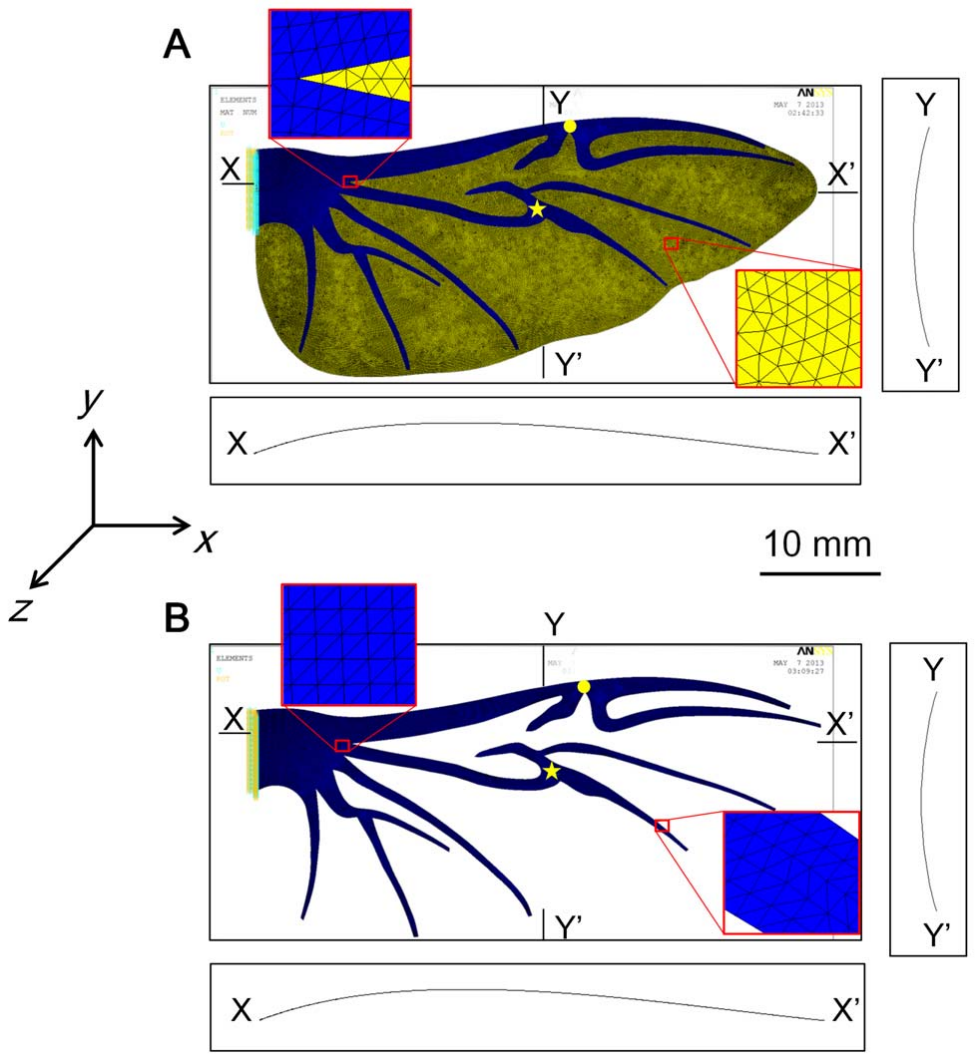
Finite element models based on the *Allomyrina dichotoma* beetle's hind wing. (A) Chord-spanwise camber intact model. (B) Chord-spanwise camber cutting model. The membrane elements are yellow and the vein elements are blue. The circle and star indicate the MJ and MVJ, respectively. The displacement is applied to MJ and MVJ. XX' and YY' show two cross sections of the model.

- No-camber model: This model has an accurate geometry of the wing but neglects the initial curvature or camber of the wing. All points on the wing lie on the *xy* plane.
- Chordwise camber intact model: The model is identical to the no-camber model except that it includes a chordwise camber of 8%. Note that the magnitude of the camber does not affect the direction of the bending asymmetry. The maximum camber of the wing at rest with a 50% wingspan is approximately 8% (camber of section XX' in Figure 1C). In addition, the maximum positive camber deformation of the hind wing during the downstroke is approximately 16% [30]. Therefore, a chordwise camber of 8% was selected for this study as an average value of the dynamic cambers of the hind wing. This model is used to investigate the effect of the camber on the bending asymmetry.
- Spanwise camber model: This model is identical to the no-camber model except that the spanwise camber of a 5.5% camber is added. The spanwise camber of 5.5% was selected from Figure 1C. This model is used to investigate the effects of the spanwise camber on bending asymmetry.
- Chord-spanwise camber intact model: The model is the same as the chordwise camber intact model, but it has a spanwise camber of 5.5%. This model is close to the real model of the wing (see Figure 2A).
- Chord-spanwise camber cutting model: This model is the same as the chord-spanwise camber intact model, but it has the membrane removed. This model is used to explore the effects of the membrane on the bending asymmetry (see Figure 2B).

In this study, we only focused on the effects of the camber and stress stiffening of the membrane. Hence, the modulus of the membrane will not affect the direction of the bending asymmetry. Thus, it was assumed that the membrane material is isotropic and homogenous with a modulus of 3.8 GPa, which represents the average modulus of the membrane [32]. The membrane was assumed to have a constant thickness of 4.5 μm (i.e. the mean thickness of the membrane at part A in Reference [32]) and was meshed with a thin shell element (SHELL181). The modulus of the vein material was measured to be 11 GPa [33]. In reality, the thickness of the vein varies from root to tip and from the leading edge to the trailing edge. The maximum and minimum values of the vein thickness were 715 and 18 μm, respectively [33]. In this study, we assumed the veins had a uniform thickness of 310 μm, which is the mean thickness of the vein from Reference [33]. The vein material was assumed to be isotropic and homogenous. The veins were meshed using a thin shell element (SHELL181). The Poisson's ratios of the vein and membrane did not affect the bending results [14]. Therefore, a Poisson's ratio of 0.3 was applied to the membrane and vein materials.

During flight, the insect wings were subjected to aerodynamic and inertial pressure. Instead of pressure loading, the point force has been used frequently in experiments and FEM to evaluate the insect wing structure performance [14]. In this study, we considered two types of loads: point force and pressure load. For the point force, forces were applied to two interesting points: the marginal joint (MJ) and the movable vein joint (MVJ). There were two reasons for selecting MJ and MVJ as the point force points. The first reason is that the real hind wing has been folded and unfolded at MJ and MVJ in experiments [34]. Thus, if forces were applied to an area other than these positions, it will bend unstably and fold at these positions. In order to avoid this phenomenon, the forces should be applied at the points from wing root to the MJ and MVJ. Note that the forces applied at MJ and MVJ will produce the largest moment arm. The second reason is that the membrane of the real hind wing is very thin and easily broken when a point force is applied. Therefore, forces were only applied at the wing veins in order to avoid breaking the wing. Moreover, MJ and MVJ are positioned on two main veins; thus, they are interesting points for the force application. The wing model was fixed at the wing root with zero displacement and rotation, and the displacement was applied to the MJ or MVJ points from the dorsal side and ventral side. ANSYS^®^ calculated the reaction force due to this displacement. Then, we compared the reaction force from the dorsal side with that from the ventral side to explain the bending asymmetry of the wing.

**Figure 3:**
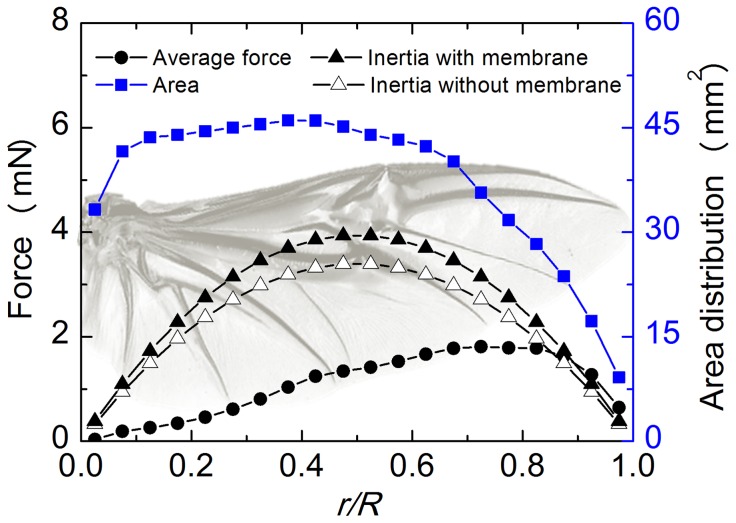
Force and area distribution along a single wingspan. The average force included the aerodynamic and inertial forces during a flapping cycle were considered as a reference force to investigate the bending asymmetry of the wing.

For the pressure load, the pressure was applied over the wing area. The magnitude of the pressure was determined based on the force distribution over the wingspan, which was estimated using the unsteady blade element theory [35]. Similar to previous studies [35], the aerodynamic forces were included through three force components: the force produced by the flapping motion of the wing (translational forces), the reaction forces of the air, which is accelerated by the wind on the wing (added mass force), and the rotational force due to the rotational circulation (rotational force) [2], [35]. The inertial forces are resulted from acceleration of wing mass. Similar to Jongerius and Lentink's work [21], we considered the inertial force due to the wing translation and ignored the inertial forces due to wing rotation. A detailed calculation of those forces is presented in the supporting information section (see File S1). The maximum inertial forces with/without membrane and the average force generated by a hind wing during a flap cycle were estimated as shown in Figure 3. We can see that the inertial force of the wing does not change too much when the membrane of the wing was removed. This can be explained that the mass of the membrane is too small compared to that of the veins. In this study, the average force included the aerodynamic and inertial forces was considered as a reference force to investigate the bending asymmetry of the wing. The maximum average force was found at approximately 80% of the wingspan (*R*). The shape of the estimated average force of the beetle in this study is similar to that of other insects such as dragonflies [21]. In order to determine the pressure, the area distribution along the wingspan was measured and is also presented in Figure 3. The area decreases sharply from root to tip at 60% of the wingspan. From the average force and area, we can estimate the pressure distribution along the wingspan.

Following the work of Combes and Daniel [14], for the pressure loading, we constrained the node at the wing base of the wing model, three translations, and two rotations so that it can rotate in the dorsal-ventral direction (around the *y*-axis). Then, we compared the deformation shape as the pressure was applied from the dorsal side and ventral side in order to explain the bending asymmetry of the wing.

### Mesh generation

Parabolic order elements were used to mesh the model because they have mid-side nodes, which can interpolate curved edges well. The wing veins were meshed using R-trias (triangle) elements due to its complex geometry, while the membrane was meshed using trias elements. The mesh was refined until the solution converged. In order to determine the minimum number of elements necessary to capture the bending behavior of the wing, we meshed the model with elements with sizes of 0.3, 0.25, 0.2, 0.15, and 0.1, and found that element size 0.1 was sufficient to ensure the asymptotic performance of the model.

### Model solution

Linear and nonlinear solutions with large deformations were applied. For the point force loading, each model was subjected to nodal displacements of 0.5 and 1 mm at two positions (MJ and MVJ) on both sides of the wing (dorsal and ventral sides). The reaction forces were calculated in order to investigate the asymmetric bending. For the pressure loading, the positive pressure applied to the wing was considered because the wing was subjected to a pressure load on the dorsal side. The negative pressure applied to the wing was considered because the wing was subjected to a pressure load on the ventral side.

## Results

### Stress stiffening effects

**Figure 4:**
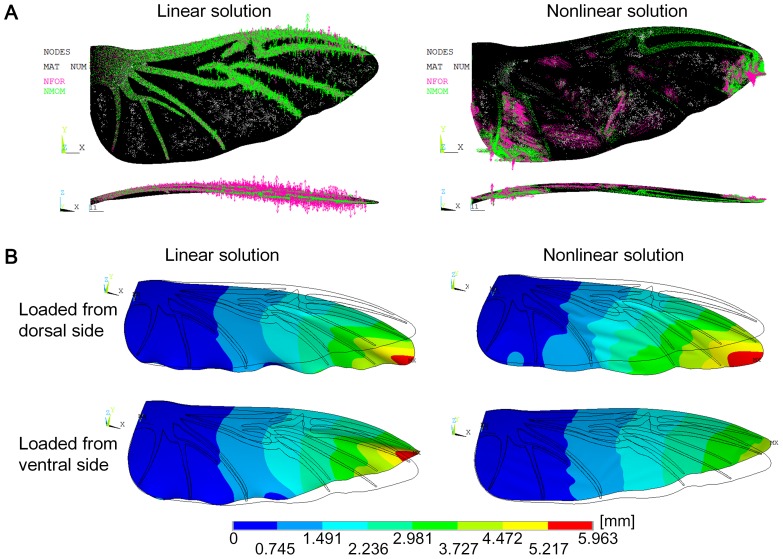
The stress stiffening effects on the chord-spanwise camber intact model. (A) The free body forces (NFOR) and moments (NMOM) in the ANSYS^®^ model in the linear and nonlinear solutions. The NFOR is indicated using pink, and the NMOM is green. (B) Displacement contour when pressure was applied to the dorsal and ventral sides in the linear and nonlinear solutions.

We conducted finite element (FE) analyses for five models with two options that were provided in ANSYS^®^: a linear solution with a small displacement static and a nonlinear solution with a large displacement static. In order to determine how the stress stiffening effects operate in the two options, we investigated the free-body forces (NFOR) and moments (NMOM) in the ANSYS^®^ models. For example, for the pressure loading on the chord-spanwise intact model, the linear solution demonstrated that the free-body forces and moments concentrated on the wing veins and the out-of-plane force components dominated the shear components (see Figure 4A). In contrast, the nonlinear solution demonstrates that the free-body forces and moments concentrate on both the wing veins and membrane, and the shear components dominated the out-of-plane components (see Figure 4A). The shear force components on the membrane have a stress stiffening function in the wing. Therefore, the nonlinear solution produces the stress stiffening in the membrane.

From the displacement results of the six models, we found that the linear solution with a small displacement static option did not produce the bending asymmetry of the wing, while the nonlinear solution with a large displacement static produced the bending asymmetry in the camber intact models (spanwise camber intact model, chordwise camber intact model, and chord-spanwise intact model). Moreover, for the chord-spanwise camber intact model, the displacement contours that result from the pressure load with the linear and nonlinear solution (see Figure 4B) demonstrated that the linear solution produced the same displacement when the pressure was applied to the dorsal side and the ventral side. The nonlinear solution produced different displacements in which the displacement for the pressure applied to the dorsal side was larger than that when the pressure was applied to the ventral side. This indicates that the stress stiffening effect has an important function in the bending asymmetry of the wing.

### Effects of camber

As stated above, only the camber intact models (spanwise camber intact model, chordwise camber intact model, and chord-spanwise intact model) with stress stiffening produced the bending asymmetry. In order to investigate the camber effect on the bending asymmetry, we compared the structure of the camber intact models and the no-camber model. For the no-camber model, all points on the wing lay in the *xy* plane; thus, this model was symmetric when the force was applied along the positive and negative *z* directions. Therefore, there was no difference in the deformation of the wing as the force was applied to the dorsal and ventral sides. As a result, the no-camber model failed to produce dorsal/ventral bending asymmetry. For the camber intact models (spanwise camber intact model, chordwise camber intact model, and chord-spanwise intact model), the wing structures were asymmetric with respect to the *xy* plane. Therefore, the camber intact models tended to produce different deformations when the force was applied along the positive and negative *z* directions. However, the camber (chordwise and spanwise camber) effect on the bending asymmetry will be investigated in the following sections.

#### Effects of chordwise camber

**Figure 5:**
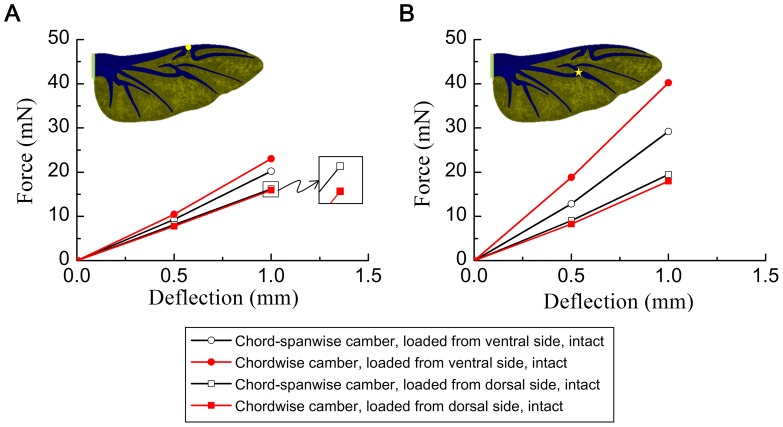
Finite element results for the force versus deflection of the chordwise camber intact model and chord-spanwise intact model at two load positions. (A) Load applied at MJ. (B) Load applied at MVJ.

In order to investigate the effect of chordwise camber on the bending asymmetry of the wing, the chordwise and chord-spanwise camber intact models were considered. The force/deflection curves of the two models are illustrated in Figure 5. These results demonstrate that the wing was significantly more rigid in loading from the ventral side than from the dorsal side. The deflections were slightly nonlinear with the force over the range used. A large asymmetry was observed as the applied force was increased on the wing. The asymmetry was larger when the force was applied to the MVJ than when it was applied to the MJ. Comparing the chordwise and chord-spanwise camber intact models, we found that the chordwise camber intact model was more rigid than the chord-spanwise camber intact model when the forces were applied from the ventral side, and the chordwise camber intact model was weaker than the chord-spanwise camber intact model when the forces were applied from the dorsal side (see Figure 5). In addition, the chord-spanwise camber intact model had less bending asymmetry than the chordwise camber intact model. Therefore, the spanwise camber affects the bending asymmetry. The effect of the spanwise camber on the bending asymmetry will be investigated in the following section.

**Figure 6:**
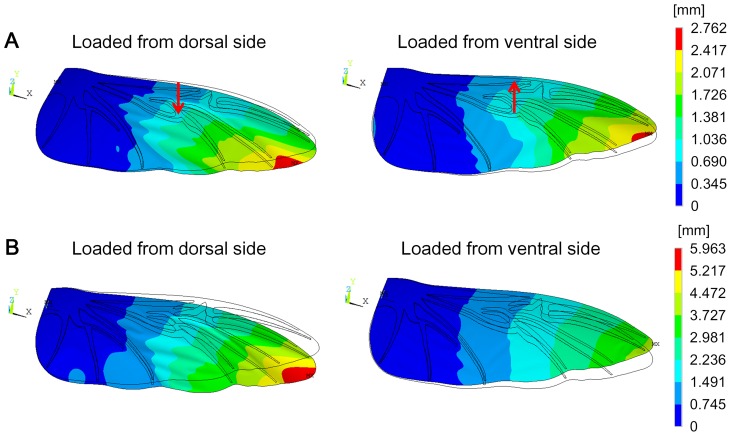
Deformations of the chord-spanwise camber intact model. (A) Deformations of the wing under 1 mm displacement applied to the MVJ (red arrow). The black lines without contours indicate the undeformed shape of the wing. The black lines with contours indicate the deformed shape of the wing. (B) Deformations of the wing due to pressure.

In order to visualize the bending asymmetry, the contour deformation was considered. Figure 6A presents the results of the deformation of the wing under 1 mm deflection applied to the dorsal and ventral sides at the MVJ position for the chord-spanwise camber intact model. At the same applied displacement, the deformation of the wing under the force applied to the dorsal side was higher than that under the force applied to the ventral side. Similar to the point force, when the wing was subjected to a pressure load from the dorsal side, the wing deformed more than when pressure was applied to the ventral side (see Figure 6B).

#### Effect of spanwise camber

**Figure 7:**
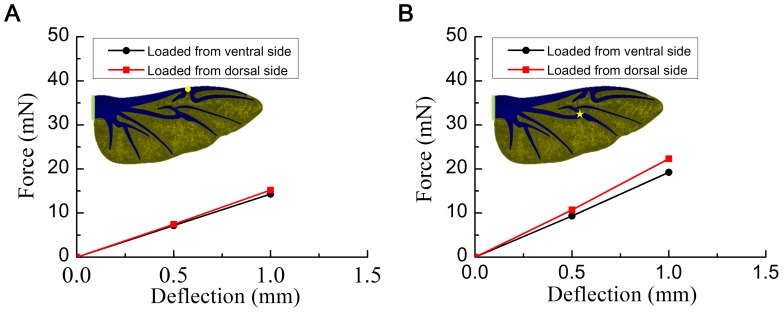
Finite element results for the force versus deflection of the beetle hind wing for the spanwise camber intact model. (A) Load applied at MJ. (B) Load applied at MVJ.

Figure 7 shows the load/deflection curves for the spanwise camber intact model. The load applied to the dorsal side was higher than that applied to the ventral side, particularly when the load was applied at the MVJ position. This indicates that the spanwise camber increased the rigidity of the wing when the load was applied to the dorsal side and weakened the wing when the load was applied to the ventral side. Therefore, the added spanwise camber in the chordwise camber intact model weakened it compared with the original model (chordwise camber intact model) when the force was applied to the ventral side and increased its rigidity compared with the original model when the force was applied to the dorsal side. This is used to explain why the chord-spanwise camber intact model is weaker than the chordwise camber intact model when the force was applied to the ventral side and more rigid than the chordwise camber intact model when the force was applied to the dorsal side (results from Figure 5).

### Role of membrane

**Figure 8:**
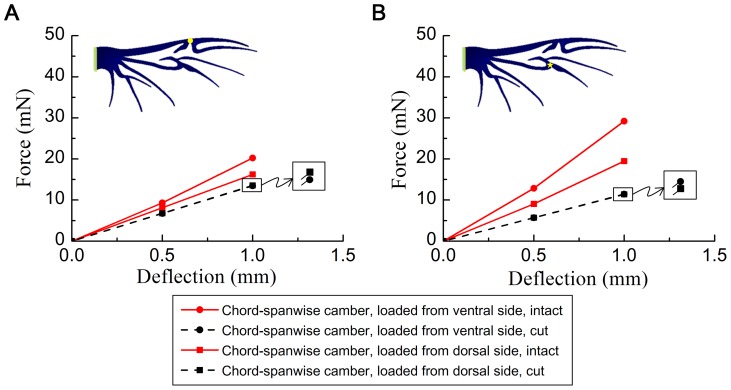
Finite element results for the force versus deflection of the chord-spanwise camber cutting model at two load positions. (A) Load applied at MJ. (B) Load applied at MVJ.

The force/deflection curves for the chord-spanwise camber cutting model are illustrated in Figure 8. The reaction forces did not change when the load was applied to the dorsal and ventral sides. This indicates that the membrane has an important function in the asymmetric bending. In addition, the force applied to the beetle's hind wing was significantly reduced when the membrane was cut from the wing. This indicates that the membrane can carry the load.

## Discussion

### Stress stiffening effects

The stress stiffening effect that is investigated in this study has been studied previously by Newman and Wootton [36]. This effect also occurs on the pleating of dragonfly wings. The cross-veins of a wing act as stiffeners within the girders, which allow the membrane to carry the web shearing forces as pure tension. Therefore, when a wing is bent, the out-of-plane stiffness of the structure can be significantly influenced by the state of the in-plane stress in the wing. That is, the membrane under tension becomes stiff in the transverse direction. This effect is important in a thin structure, especially in a thin cambered plate such as an insect wing. Stress stiffening effect was first considered in the insect wing model [16]. In this study, the FEM solution with stress stiffening exhibited the bending asymmetry while the FEM solution without stress stiffening did not.

The linear solution with a small displacement (without stress stiffening) failed to produce the bending asymmetry in the wing. This result is explained using the basic equation of the finite element method [*K*]{*u*}  =  {*F*}, where [*K*] is the stiffness matrix, {*u*} is the displacement vector, and {*F*} is the applied load vector. In the small displacement theory, the stiffness matrix is constant and is based on the initial undeformed position geometry of the wing [22]. Moreover, the initial undeformed position is the same despite the load being applied to the dorsal side or the ventral side; therefore, there is no bending asymmetry. In contrast, for the nonlinear solution with a large deflection, in which stress stiffening is included, the stiffness matrix is recalculated at each deformed position of the wing. When the wing deforms, the second moment of the wing cross section will change. Therefore, if the second moment of the wing cross section tends to increase, the stiffness will increase. This leads to the force increasing.

In the no-camber model with a nonlinear solution, the cross section was a line (not a curve) that was symmetric with respect to the *xy* plane. The cross section changed in the same manner at each deformed position when the load was applied to the dorsal and ventral sides. Therefore, at each deformed position, the second moment of the cross sectional area of the wing when the load was applied to the ventral side was similar to that of the wing when the load was applied to the dorsal side. Hence, this model failed to produce the bending asymmetry.

### Effects of camber

**Figure 9:**
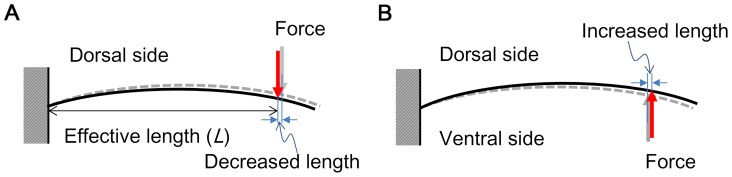
Schematic of the spanwise camber intact model under a load. (A) Load on the dorsal side, where the force tends to decrease the effective length. (B) Load on the ventral side, where the force tends to increase the effective length. The curve in the figure shows the spanwise camber. The dashed lines indicate the initial position; the solid lines indicate the deformed position.

In the spanwise camber intact model with a nonlinear solution, the force applied to the dorsal side was higher than that applied to the ventral side. This indicates that the wing was more rigid when the load was applied to the dorsal side than when it was applied to the ventral side. To explain this result, we use the beam theory: we considered the wing to be a beam. In the loaded from dorsal side case, the force tended to increase the spanwise camber, which decreases the effective length (*L*) (the distance between the point force and the clamp), while in the loaded from ventral side case, the force tended to decrease the spanwise camber, which increased the effective length (see Figure 9). Therefore, with the same applied deflection, the force applied to the wing, which was calculated as *F* = 3*EIδ/L* ^3^ [20], will be larger when the force is applied to the dorsal side compared with when it is applied to the ventral side because the term *EIδ* is constant. (For the spanwise camber model only, the cross section is symmetric in the dorsal-ventral plane: the moment of inertia (*I*) is constant; Young's modulus (*E*) is constant; and the same displacement (*δ*) was applied). However, the difference in force between the dorsal side and ventral side was small compared with that in the case of the chordwise camber intact model. A higher dorsal bending rigidity was also found in the anal fan (part A – the leading edge spar) of a locust hind wing [15], which is in agreement with this explanation.

In the chordwise camber intact model with a nonlinear solution, when the load was applied to the ventral side, the force tended to increase the camber and increase the second moment of the cross sectional area of the wing. In contrast, when the load was applied to the dorsal side, the force tended to flatten the camber and reduce the second moment of the cross sectional area. Therefore, with the same applied deflection, the second moment of the cross sectional area of the wing when the load was applied to the ventral side was larger than that when the load was applied to the dorsal side. This stiffens the wing when the force is applied to the ventral side. This observation is similar to the findings of Wootton *et al*. [19], [37].

**Figure 10:**
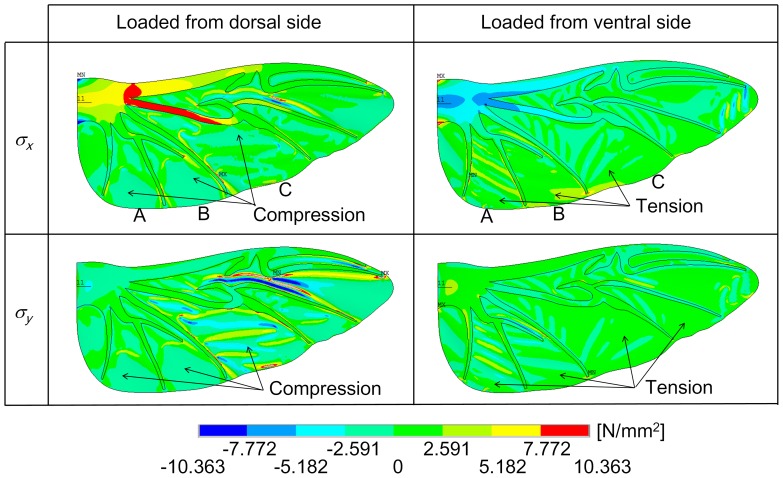
Contour plot of the normal stresses in the *x* and *y* directions (*σ_x_* and *σ_y_*) due to the pressure load in both sides (dorsal and ventral sides) of the chord-spanwise camber intact model.

**Figure 11:**
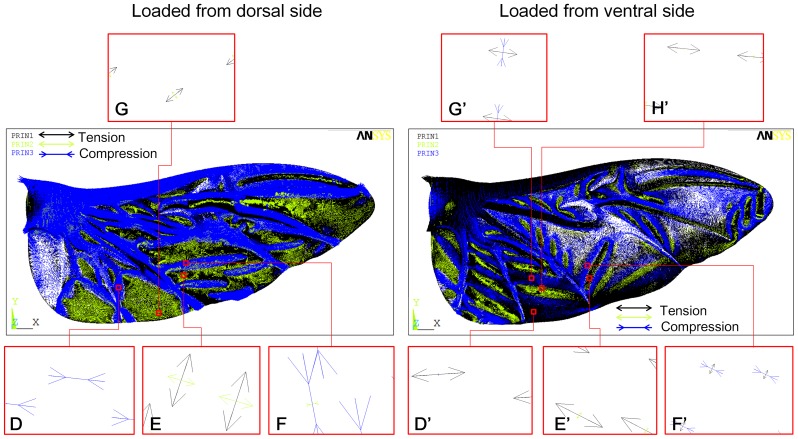
Orientation of the principal stresses (*σ_1_*, *σ_2_*, and *σ_3_*) due to the pressure load on both sides (dorsal and ventral sides) of the chord-spanwise camber intact model.

In order to understand the behavior of the wing under the force applied to the dorsal and ventral sides more thoroughly, the stresses that result from the load of the wing were investigated. For example, we considered the stress distribution of the wing under pressure load applied to the dorsal and ventral sides of the chord-spanwise camber intact model as shown in Figures 10 and 11. Figure 10 shows the stress tensor components (normal stress in the *x* direction (*σ_x_*) and normal stress in the *y* direction (*σ_y_*)). To analyze this easily, we separated the wing membrane into small regions (A, B, and C) as shown in Figure 10. It is clear to see that when the force was applied to the ventral side, the membrane was in tension in both directions (*x* and *y* directions). The tensions in membrane constrain the veins (as seen in regions A and B). Therefore, the membrane can carry the load, which leads to a more rigid wing than when the force is applied to the ventral side. In contrast, when the force was applied to the dorsal side, the membrane was compressed in both directions (*x* and *y* directions), particularly in regions A and B. The compressed membrane indicates that the membrane does not carry the load. Therefore, the wing is weak when the force was applied to the dorsal side.

Figure 11 shows the orientation of the principal stresses (*σ_1_*, *σ_2_*, *σ_3_*). When the load was applied to the dorsal side, the third principal stress (*σ_3_*) (compression stress) dominated the other stresses (*σ_1_* and *σ_2_*). The third principal stress (*σ_3_*) orients from the root to the tip (see Figure 11D), whereas the first and second stresses (*σ_1_* and *σ_2_*) tend to orient from the leading edge to the trailing edge (see Figures 11E, G). The orientation of *σ_3_* tends to reduce the effect of the veins in the load bearing capacity. Hence, the wing is weaker when the force was applied to the dorsal side. When the load was applied to the ventral side, the first principal stress (*σ_1_*) (tension stress) dominated the other stresses (*σ_2_* and *σ_3_*). The first principal stress (*σ_1_*) almost orients from the root to the tip (see Figures 11D', G', H'); thus, it constrains the two neighboring veins together. This increases the wing bearing capacity. In conclusion, the chordwise camber increased the rigidity of the wing when the load was applied to the ventral side.

### Role of membrane

The chord-spanwise cutting models were more flexible compared with the intact model. This flexibility demonstrated that the membrane contributed significantly to the bending rigidity of the wing. In the intact model, when a force was applied to a wing, it was transmitted through the veins in the form of tension or compression. This causes the wing to increase or reduce the load bearing capacity. In contrast, in the cutting model, when a force was applied to a wing, the veins carried the load independently and the stress stiffening effect disappeared. Therefore, the capacity of the carrying load was similar when the load was applied to the dorsal and ventral side.

In this study, we used the following assumptions. The veins were assumed to have a uniform thickness from the root to the tip, and their cross sections were modeled as a rectangle. The membrane was assumed to have the same material properties and thickness. However, in reality, the cross section of the vein is hollow, and the vein tapers from the root to the tip. Moreover, the veins of the wing are linked by a membrane whose thickness and properties vary around the wing [32]. In addition, the camber was assumed to have a constant value of 8% in the FEM model, although the camber of a real wing does not have a constant value but rather it varies from the root to the tip. In this study, we only focused on the effects of the camber and stress stiffening of the membrane; thus, the assumptions about the camber and geometry do not affect the direction of the bending asymmetry. Based on the theoretical and experimental results, Song *et al.* [38] found that the maximum deflection of the compliant membrane wing is a function of aerodynamic loading. Therefore, the direction of the bending asymmetry is independent of the amplitude of applied pressure.

The present investigation confirmed the significant functions of the camber and the stress stiffening effects in the membrane on the dorsal/ventral bending asymmetry of insect wings. It has provided a potential approach to a more rigorous analysis of thin, complex, and delicate bio-structures.

## Conclusions

In this study, the asymmetric bending of the *Allomyrina dichotoma* beetle's hind wing was successfully investigated through FEM analyses. Five different finite element models of the *Allomyrina dichotoma* beetle's hind wing (no-camber model, chordwise camber intact model, spanwise camber intact model, chord-spanwise camber intact model, and chord-spanwise camber cutting model) were modeled using the ANSYS^®^ software. These models were subjected to displacement loadings and pressures from the dorsal side and ventral side. The results revealed that both the stressed stiffening of the membrane and the camber of the wing affect the bending asymmetry of insect wings. In particular, the chordwise camber increased the rigidity of the wing when the load was applied to the ventral side, while the spanwise camber increased the rigidity of the wing when the load was applied to the dorsal side. These results provide a comprehensive explanation on the mechanical behavior of cambered insect wings, including the bending asymmetry behavior, and provide a potential approach to a more rigorous analysis of thin, complex, and delicate bio-structures.

## Supporting Information

File S1 (DOCX)
