# Supplementary material for: Biomechanical Properties of Insect Wings: The Stress Stiffening Effects on the Asymmetric Bending of the Allomyrina dichotoma Beetle's Hind Wing
Source: PLoS One. 2013 Dec 5;8(12):e80689. doi: 10.1371/journal.pone.0080689 (PMC3855175; doi:10.1371/journal.pone.0080689)
Supplement: File S1 — (DOCX) [file pone.0080689.s001.docx]

**Supporting Information File S1**

**Blade element theory for aerodynamic and inertial forces estimation**

The wing kinematics of freely flying beetle that can be found in Truong’s work [1] is briefly presented here. The wing kinematics of the beetle with markings on its wings was ﬁlmed using three high-speed cameras (Photron Ultima APX, 1024×1024 pixels, Japan) at 2000 frames per second. The direct linear transformation (DLT) method which has been programed in MATLAB® (V.7.0, MathWorks Inc., USA) application by Hedrick [2] to analyze the movies of the free-ﬂying beetle. By using the program and the sequential images containing the markings on the wing, the ﬂapping angle and the rotational angle of four wing sections at four positions (20%, 40%, 60% and 80% wingspan) were determined. The stroke plane was about 30^o^. The ﬂapping frequency of the beetle was approximately 37.7 Hz. Time histories of the ﬂapping angle and the rotational angle of the four wing sections are plotted in Figure S1

Figure S2(A) shows a flapping wing of length *R*. In order to describe the flapping motion of the wing, we defined an orthogonal coordinate system (*Oxyz*) as shown in Figure S2(A). According to Truong *et al*. [1], the translation force in the *ζ* direction, which is perpendicular to the plane stroke, is calculated using the following equation:

$F_{T\zeta}=\frac{1}{2}\rho\int_{0}^{R} c\left[C_{L}V_{t}-C_{D}V_{i}\right]\sqrt{V_{i}^{2}+V_{T}^{2}}\text{d}r$, (1)

where *ρ* is the density of the air, *C_L_* and *C_D_* are the measured lift and drag coefficients presented in Reference [3], respectively, *V_T_* is the translational velocity, , *V_i_* is an induced velocity, c(*r*) and d*r* are the chord length and width of a wing section, respectively.

The added mass forces acting on the wing are calculated by:

$F_{T\zeta}=-\frac{\pi}{4}\rho\int_{0}^{R} c^{2}a_{n}\sin\theta\text{d}r$, (2)

where *a_n_* is the acceleration of a reference point that represents the motion of the mass (d*m*) in the direction perpendicular to the surface of the wing section. The acceleration (*a_n_*) of the reference point is determined as follows:

, (3)

where *r* is the distance from the wing hinge and *θ_r_* is the rotation angle, which is defined as the angle between the *η*-axis and the chord of the wing section, as shown in Figure S2(B). *ψ* is the flapping angle. *x_m_* is the distance from the reference point to the rotation axis.

The rotation forces acting on the wing are calculated by:

$F_{rot,\zeta}=\int_{0}^{R} \text{d}F_{rot}\cos\theta_{r}$, (4)

where d*F*_rot_ is the amplitude of the rotational force acting on a wing section and is determined using the following equations:

$\text{d}F_{rot}=\rho V_{T}\text{d}\Gamma_{\text{rot}}$, (5)

, (6)

where *c*_rot_ is the rotational force coefficient and is a function of a non-dimensional rotational velocity. The position of the feather axis is *x_f_*. Sane and Dickinson [4] predicted *c*_rot_ based on the quasi-steady condition in the following equation:

*c*_rot_ = π(0.75−*x*_f_/*c*). (7)

The inertial force of an inﬁnitesimal wing section due to the accelerating mass d*m* is estimated using the following equations:

(8)

where d*m* is the mass of an infinitesimal chord of the wing section, *ẍ*, *ӱ*, and *ẅ* are the acceleration of the mass d*m* in the *x*, *y*, and *z* direction, respectively. The acceleration *ẍ*, *ӱ*, and *ẅ* are defined as

(9)

(10)

(11)

Similar to Jounerius and Lentink’s work [5], we considered acceleration due to the wing translation and neglected accelerations due to the wing rotation. The inertial force in the *η* direction was determined as follows:

$F_{I\eta}=-F_{Ix}\sin\psi+F_{Iy}\cos\psi$ (12)

FIGURE LEGENDS

Figure S1. Wing kinematics of the beetle. Flapping angle and rotational angle

Figure S2. Blade element theory. (A) Definitions of the wing section. (B) The force components for BET analysis.

Figure S1





Figure S2


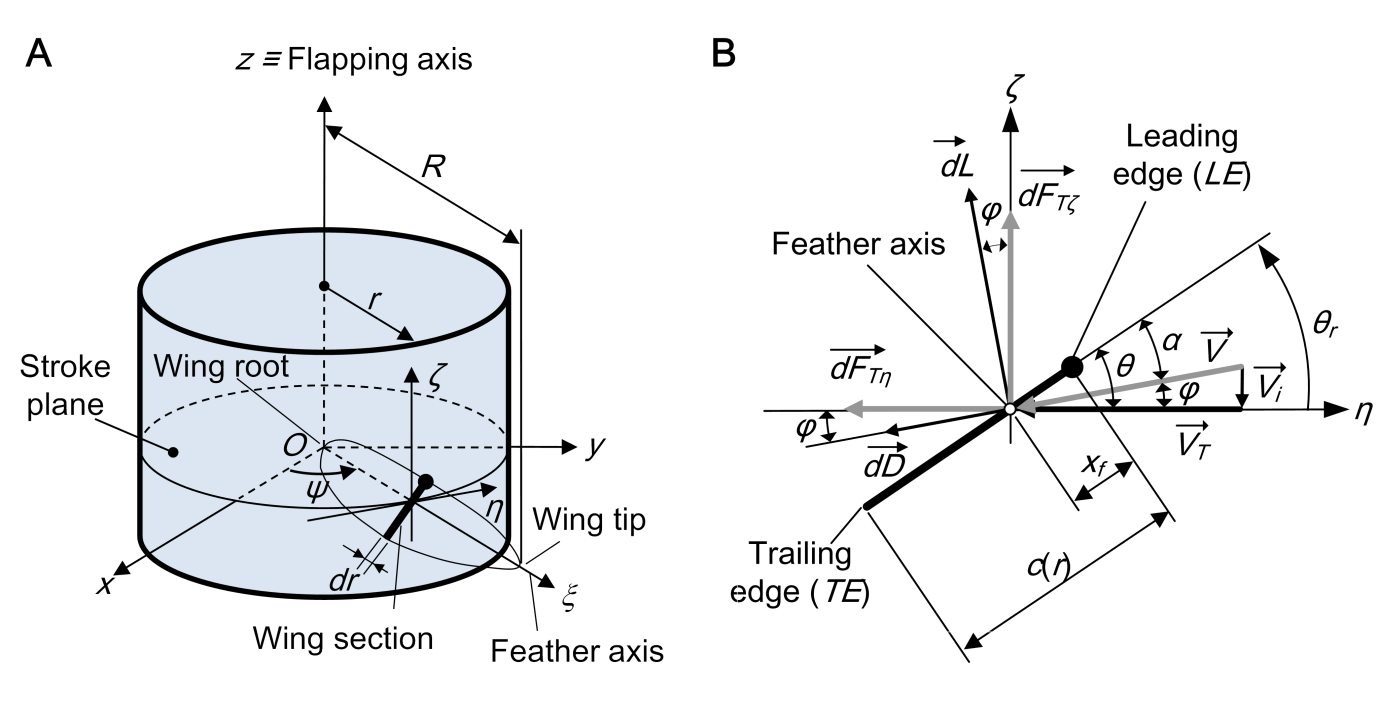


REFERENCES

1. Truong QT, Nguyen QV, Truong VT, Park HC, Byun DY, Goo NS (2011) A modified blade element theory for estimation of forces generated by a beetle-mimicking flapping wing system. Bioinsp Biomim 6: 036008−036019.
2. Hedrick TL (2008) Software techniques for two- and three-dimensional kinematic measurements of biological and biomimetic systems. Bioinspir Biomim 3: 034001-034007
3. Dickinson MH, Lehmann FO, Sane SP (1999) Wing rotation and the aerodynamic basis of insect ﬂight. Science 284: 1954-1960.
4. Sane SP, Dickinson MH (2002) The aerodynamic effects of wing rotation and a revised quasi-steady model of flapping flight. J Exp Biol 205: 1087-1096.
5. Jongerius SR, Lentink D (2010) Structural analysis of a dragonfly wing. Exp Mech 50: 1323-1334.
